# A comparison of a short versus a conventional femoral cementless stem in total hip arthroplasty in patients 70 years and older

**DOI:** 10.1186/s13018-016-0367-0

**Published:** 2016-03-22

**Authors:** Huachen Yu, Haixiao Liu, Man Jia, Yuezheng Hu, Yu Zhang

**Affiliations:** Department of Orthopaedics, Second Affiliated Hospital of Wenzhou Medical University, 109 Xueyuan West Road, Wenzhou, Zhejiang 325027 China

**Keywords:** Total hip arthroplasty, Elderly, Short femoral stem, Conventional femoral stem

## Abstract

**Background:**

The aim of this study was to compare clinical and radiological outcomes between a short femoral cementless stem and a conventional femoral cementless stem in total hip arthroplasty in patients 70 years and older.

**Methods:**

From December 2011 and July 2013, we retrospectively reviewed 50 patients (55 hips) 70 years and older treated with a short femoral cementless stem and 53 patients (58 hips) 70 years and older treated with a conventional femoral cementless stem. Their mean age was 74 ± 13.2 years and 75 ± 10.4 years, respectively. The mean follow-up was 40 ± 3.6 months and 42 ± 5.2 month, respectively. They were pre- and postoperatively evaluated by the clinical and radiological examination.

**Results:**

There was no difference in terms of average operative time, average estimated blood loss, and average hemoglobin at discharge between the short stem and the conventional stem. No patients with the short stem had intra-operative fracture, but five patients with the conventional stem had intra-operative fracture. At final follow-up, there was no statistically significant difference in Harris Hip Score, and radiographic review level between two stems. No hip with the short stem had thigh pain, but six hips with the conventional stem had thigh pain at the final follow-up. No component was revised for aseptic loosening in either group.

**Conclusions:**

Our study demonstrated that both short cementless stem and conventional cementless stem provided stable fixation and achieved a satisfactory result in patients 70 years and older and the short cementless stem had a low incidence of thigh pain and intra-operative fracture.

## Background

Total hip arthroplasty (THA) is one of the most commonly performed and successful operations in orthopedic surgery. As life expectancy continues to increase around the world, more and more elderly patients are undergoing THA. The primary goal of THA is to improve quality of life by the relief of pain. Though the efficacy of conventional femoral cementless stems has been proved to improved pain and function and overall survivorship ranging from 94 to 100 % at up to 20 years’ follow-up [1–4], there are still several disadvantages including proximal-distal mismatch, nonideal load transfer, loss of bone, thigh pain, and periprosthetic fracture [5, 6]. Short femoral cementless stems are thought to preserve more native host bone and optimize proximal load transfer, and while not a novel concept, they have become increasingly utilized in the young patients [6, 7]. Patients 70 years and older typically have a greater proportion of Dorr type B and C bone and have poor bone quality [8]. It is unclear whether patients 70 years and older would achieve stable fixation and pain relief and function from the short cementless stem. Therefore, we compared a short cementless stem to a conventional cementless stem in THA in patients 70 years and older in terms of the clinical and radiological outcomes.

## Methods

### Patients and surgery

This study was performed following the Declaration of Helsinki principles and was approved by the Institutional Review Board (IRB) of The Second Affiliated Hospital of Wenzhou Medical University. Informed consent to participate was obtained from all participants and consent to publish was obtained for the patients whose x-rays appear in this publication.

From December 2011 and July 2013, 55 patients 70 years and older who underwent 60 primary THAs with a short femoral cementless stem and 60 patients 70 years and older who underwent 68 THAs with a conventional femoral cementless stem were reviewed in this study. The indications for THA were osteonecrosis, osteoarthritis, rheumatoid arthritis, and femoral neck fracture. Of these patients, 3 died in the short group and 4 died in the conventional group secondary to causes unrelated to our THA; 2 patients in the short group and 3 patients in the conventional group were lost to follow-up. These five exclusions left 50 patients (55 THAs) in the short group and seven exclusions left 53 patients (58 THAs) in the conventional group. We compared the two groups in terms of mean ages of patients at the time of surgery, in number of hips, demographic ratios, mean body mass index (BMI), months of follow-up, diagnosis classification, and Dorr bone quality classification [8]. All data were obtained from medical records.

### Surgical technique

All patients received prophylactic antibiotics and routine postoperative thromboembolic prophylaxis with low molecular weight heparin. The same surgeon, with more than 10 years’ THA operation experience, performed all of the arthroplasties with standardized operative technique through a posterolateral approach. All hips in both groups were reconstructed with a cementless Pinnacle acetabular component (DePuy). Acetabular fixation was achieved with a press-fit technique with underreaming by 1 mm. Acetabular screws were used at the surgeon’s discretion. The median cup size was 52 mm, with a range from 46 to 60 mm. All patients in the short-stem group received Tri-locked stem (Depuy). It is made of a titanium alloy (Ti-6Al-4V) and has a cementless flat tapered wedge design. The short stem was inserted in a broach-only fashion and obtained a tight metaphyseal fit. All patients in the conventional group received Corail stem (Depuy). It is a fully hydroxyapatite coated non-porous forged titanium alloy stem and has a proximal trapexoidal cross section and a tapered distal design. All conventional stems were inserted in the reaming and broaching fashion and obtained a tight metaphyseal fit. All patients in the both groups received a ceramic femoral head from 28 to 36 mm.

All patients in both groups underwent the same postoperative protocol. They were allowed to mobilize on the second postoperative day and progress to full weightbearing with crutches as tolerated. They were advised to use a pair of crutches for 6 weeks and walk with a cane thereafter if required. All patients were able to stop using the cane in 3 months.

### Clinical evaluation

Operative times, estimated blood loss, hemoglobin level at discharge, and complications were reviewed for all patients. All patients had routine postoperative follow-up at 3 months, 1 year, and yearly thereafter. A standard physical examination was performed, including evaluation of the wound and strength assessment of the involved hip. The clinical assessment included a review of the Harris Hip Score (HHS) [9] and thigh pain at each visit. Thigh pain was defined as pain in the anterior thigh below the inguinal area [10].

### Radiological evaluation

Standardized anteroposterior and lateral radiographs were taken preoperatively, immediately postoperatively and at each subsequent visit. Two observers evaluated the radiographs. Varus and valgus angulations (the difference between the femoral diaphyseal axis and the stem’s main axis) were measured and a difference of >5° was considered varus or valgus. Definite loosening of the femoral component was defined if there was progressive axial subsidence of >3 mm or a varus or a valgus shift of >3° [11]. Bone ingrowth to the components was considered to have occurred when there was a direct contact between the trabecular bones of the femur with the components. They were classified as osseo-integrated, fibrous stable, or unstable [12]. Stress shielding was graded on the radiographs at the final follow-up according to the classification of Engh and Bobyn [13].

### Statistical analysis

All data were expressed as mean ± standard deviations. We compared the two groups for mean age, BMI, months of follow-up, operative times, estimated blood loss, hemoglobin level at discharge, preoperative and postoperative HHS, changes in HHS, and mean limb-length discrepancy using two-tailed Student’s *t* tests. We compared demographic ratios, diagnosis classification, Dorr bone quality classification, complications, thigh pain, and varus alignment using the chi-squared test. All statistical analyses were performed using the Statistical Package Social Sciences software, version 14.0 (SPSS Inc., Chicago, Illinois), and statistical significance was determined *P* < 0.05.

## Results

### Clinical results

The mean ages of patients at the time of surgery in the short stem and conventional stem groups were 74 ± 13.2 and 75.6 ± 10.4 years, respectively. The mean follow-up was 40 ± 3.6 months and 42 ± 5.2 months, respectively. Preoperative characteristics of both groups were similar in number of hips, sex, mean age, mean body mass index (BMI), Dorr classification, and disease classification (Table 1).

**Table 1:** The demographic of the two stem groups

Variable	Short stem	Conventional stem	*P* value
Number of patients	50	53	
Number of hips	55	58	
Male to female ratio	20:30	10:43	0.18
Mean age (years) (range)	74 ± 13.2	75 ± 10.4	0.320
Mean BMI (kg/m^2^)	28.1 ± 7.0	29.2 ± 6.1	0.212
Dorr bone quality			0.843
Type A	18	20	
Type B	14	12	
Type C	23	26	
Diagnosis			0.954
Osteonecrosis	11	12	
Osteoarthritis	32	33	
Rheumatoid arthritis	2	1	
Femoral neck fracture	10	12	
Follow-up (months)	40 ± 3.6	42 ± 5.2	0.620

Between the two groups, there was no difference in terms of average operative time, average estimated blood loss, and average hemoglobin at discharge. No hip in the short-stem group and five (8.6 %) in the conventional stem group had an intra-operative fracture. These were treated with cerclage wiring and healed without further complication. One patient in the short-stem group and two patients in the conventional stem group suffered a superficial wound infection that was treated successfully with superficial irrigation and debridement. Three patients in the short-stem group and six patients in the conventional stem group had a urinary tract infection that was successfully with antibiotic therapy. There was no significant difference in incidence of complication between the two groups.

All patients in both groups improved function and satisfaction with the outcome. HHS improved significantly for all patients after THA regardless of femoral stem selection (*P* < 0.01). The mean HHS improved from 47 to 85.7 for the short stem and from 55 to 86.1 for the conventional stem. However, comparing of mean preoperative and postoperative HHS, there was no significant differences between two groups. No hip with the short stem had thigh pain, but six hips (10.3 %) with the conventional stem had thigh pain at the final follow-up. There was a significant difference in the level of thigh pain between both groups (*P* < 0.05) (Table 2).

**Table 2:** Clinical results of the two stem groups

Outcome variables	Short stem	Conventional stem	*P* value
Average operative time (min)	67.4 ± 5.8	69.5 ± 7.8	0.182
Average estimated blood loss (mL)	140.2 ± 10.1	146.3 ± 8.9	0.321
Average hemoglobin at discharge (g/dL)	10.9 ± 2.4	10.1 ± 3.1	0.338
HHS			
Preoperative	47 ± 3.5	55 ± 5.2	0.516
Final follow-up	85.7 ± 4.6	86.1 ± 6.8	0.628
Thigh pain (*n*, %)	0	6 (10.3 %)	0.027

### Radiography results

**Fig. 1:**
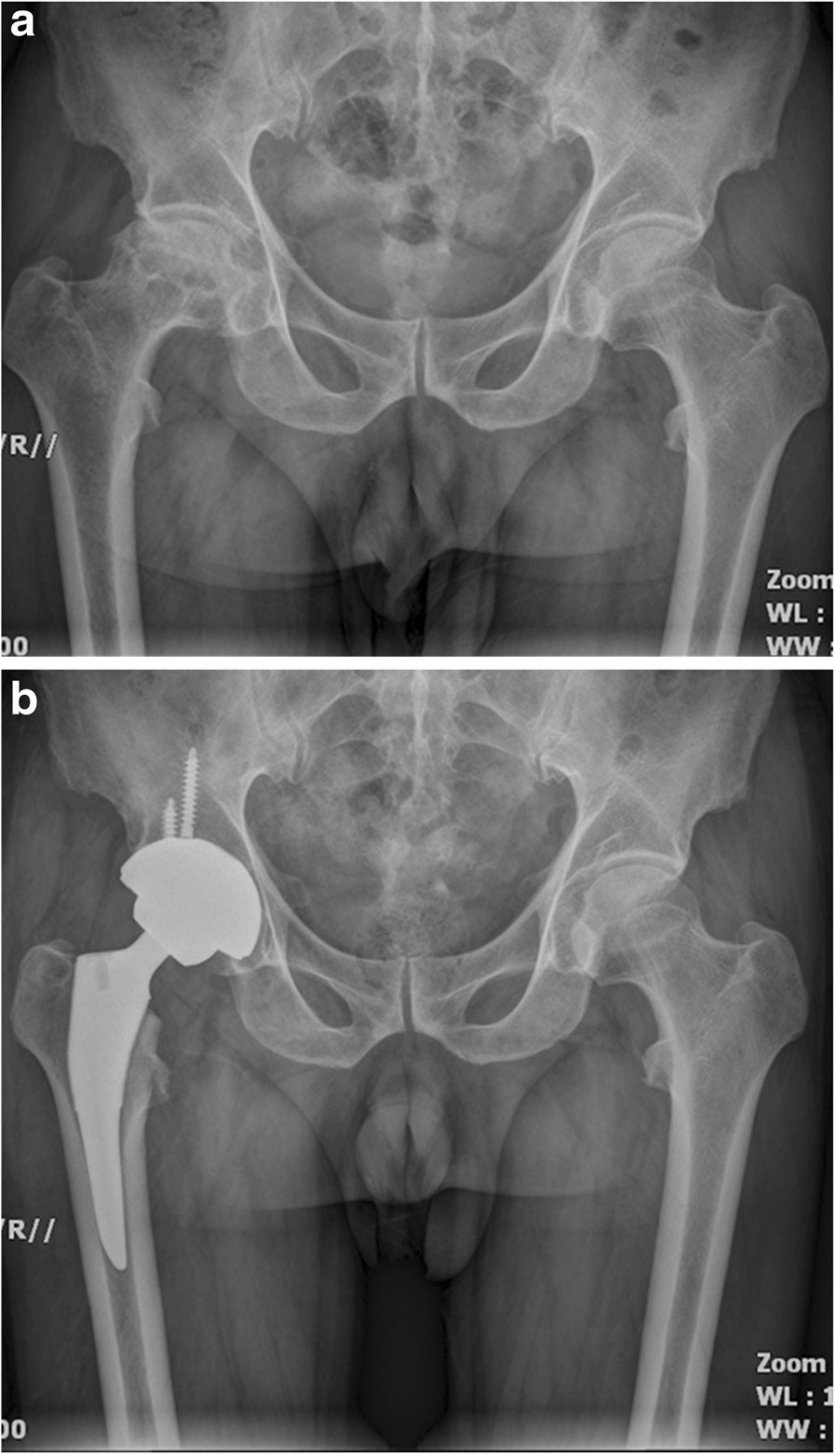
**a** A preoperative radiograph of the hip of a 75-year-old man who had femoral head osteonecrosis. **b** A radiograph at 28 months after implantation of short cementless femoral stem showed solid fixation in a satisfactory position

**Fig. 2:**
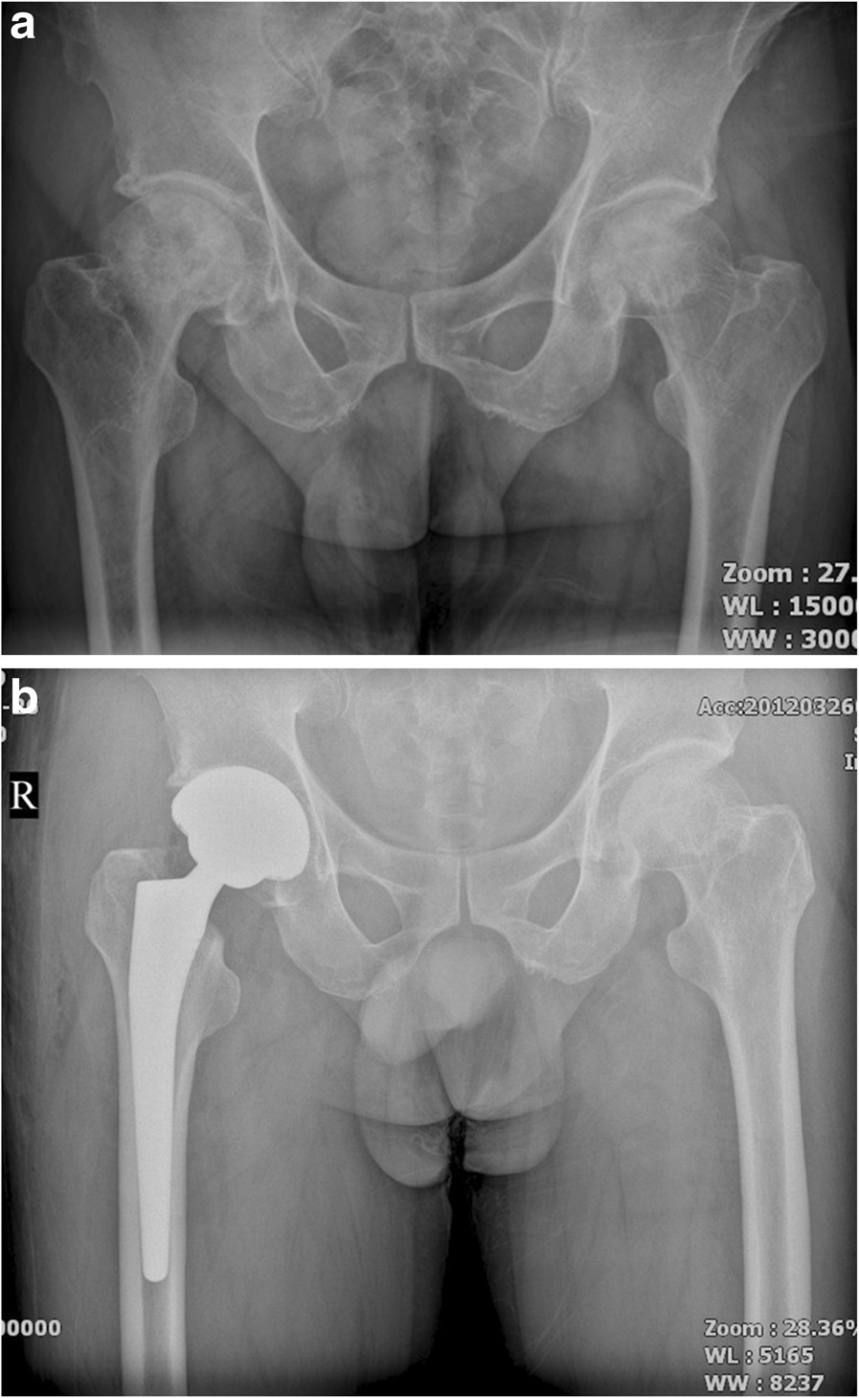
**a** A preoperative radiograph of the hip of a 73-year-old man who had femoral head osteonecrosis. **b** A radiograph at 28 months after implantation of conventional cementless femoral stem showed solid fixation in a satisfactory position

**Fig. 3:**
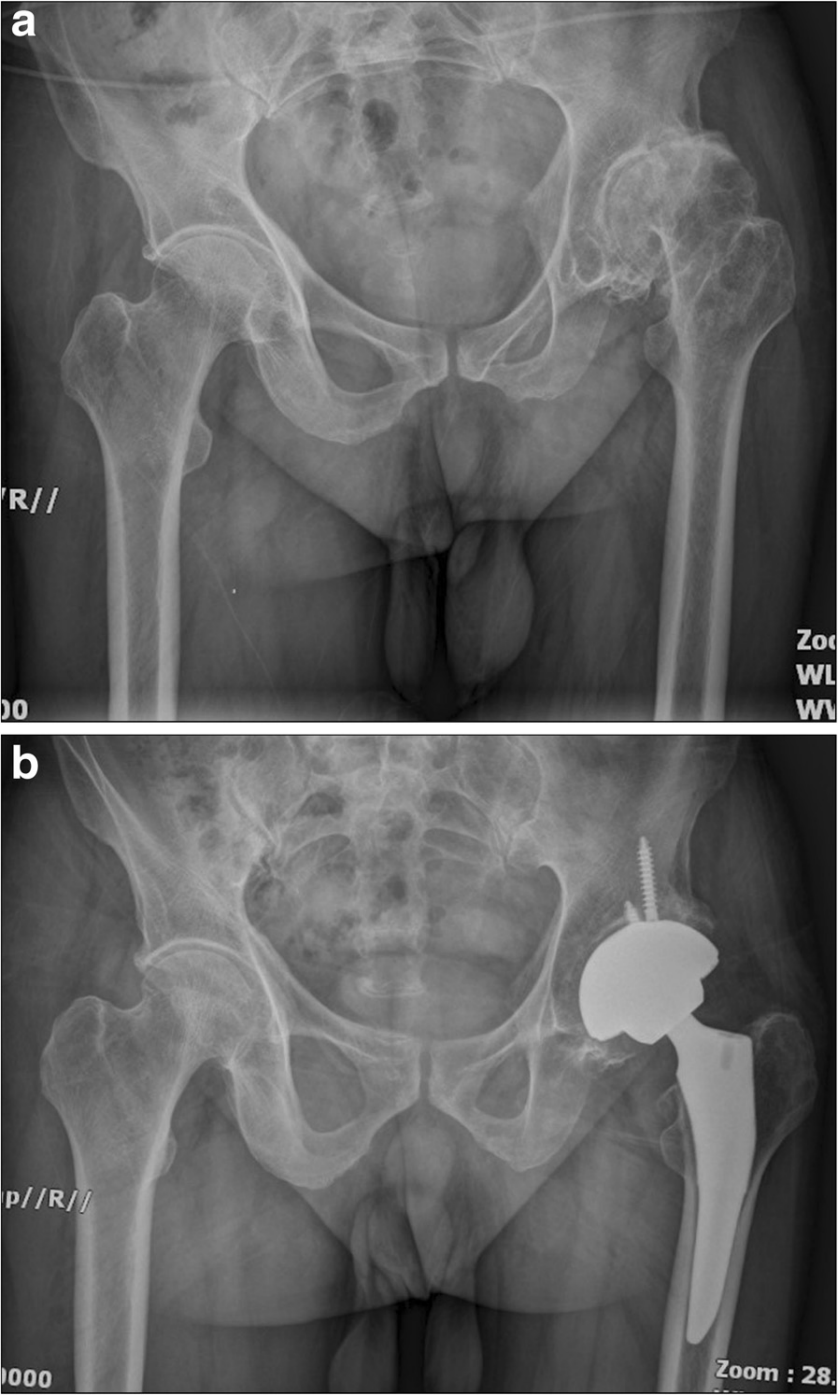
**a** A preoperative radiograph of the hip of a 73-year-old man who had left hip osteoarthritis. **b** A radiograph at 30 months after implantation of short cementless femoral stem showed solid fixation in a satisfactory position

**Fig. 4:**
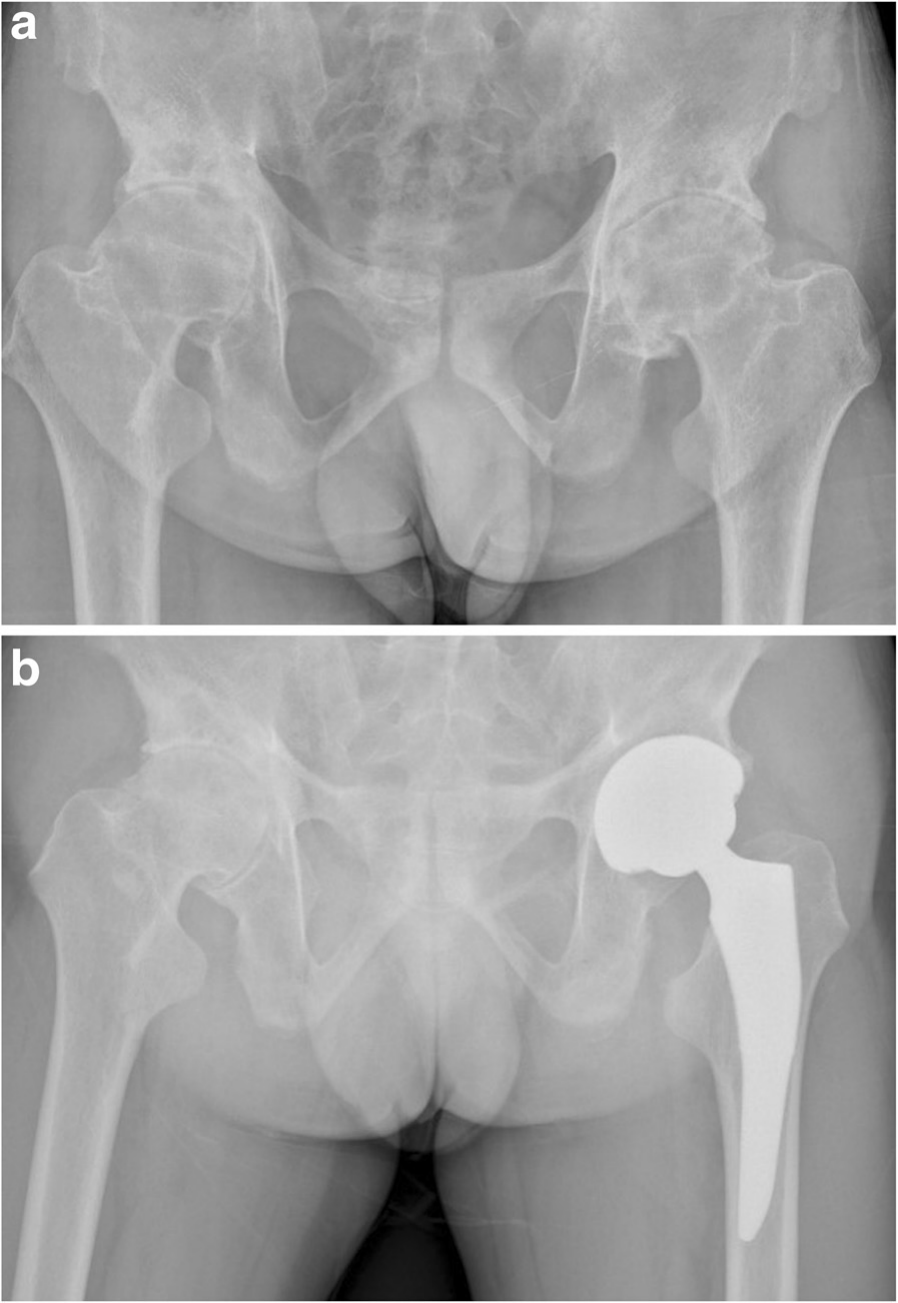
**a** A preoperative radiograph of the hip of a 73-year-old man who had bilateral hip osteoarthritis. **b** A radiograph at 38 months after implantation of short cementless femoral stem in the left hip showed solid fixation in a satisfactory position

**Fig. 5:**
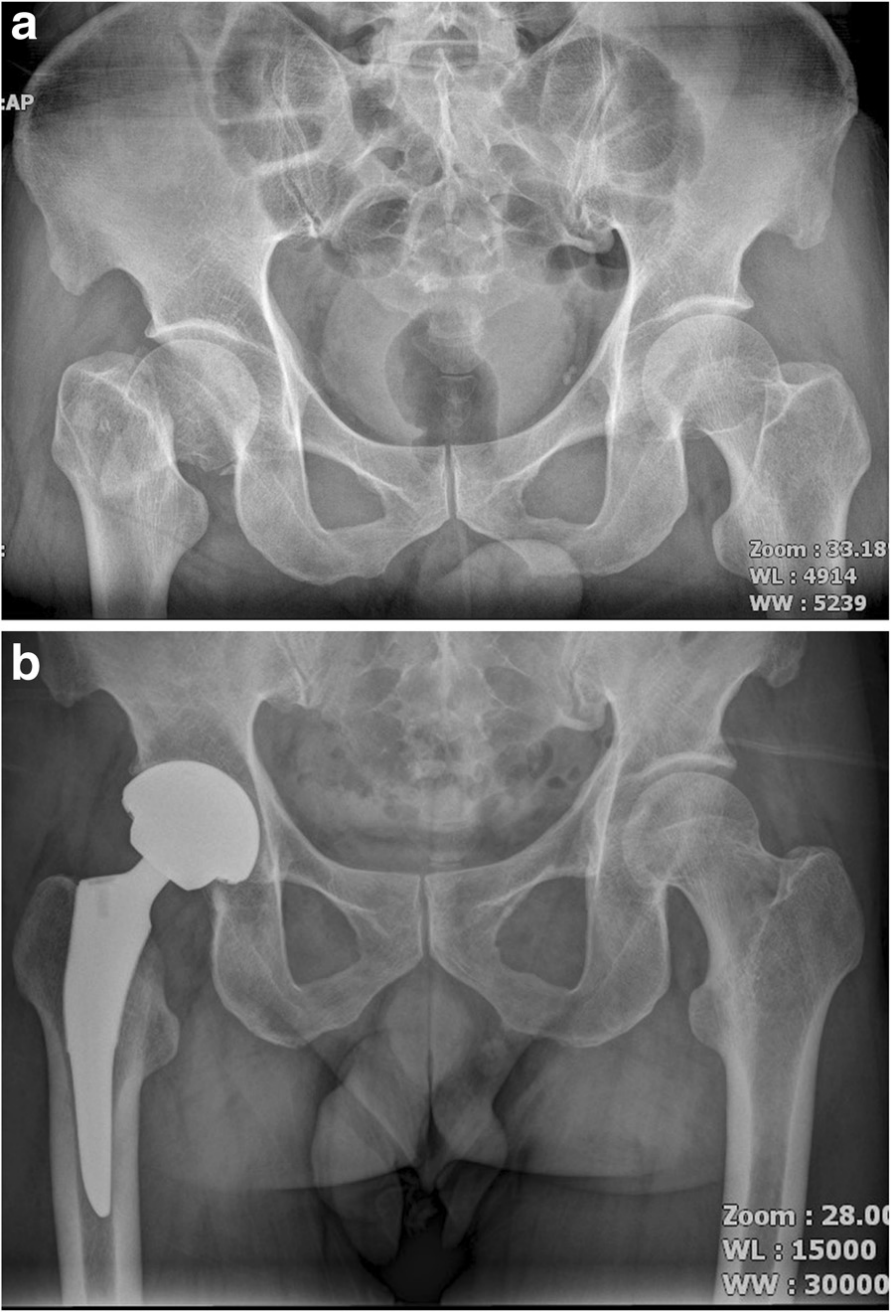
**a** A preoperative radiograph of the hip of a 73-year-old man who had right femoral head neck fracture. **b** A radiograph at 40 months after implantation of short cementless femoral stem showed solid fixation in a satisfactory position

**Fig. 6:**
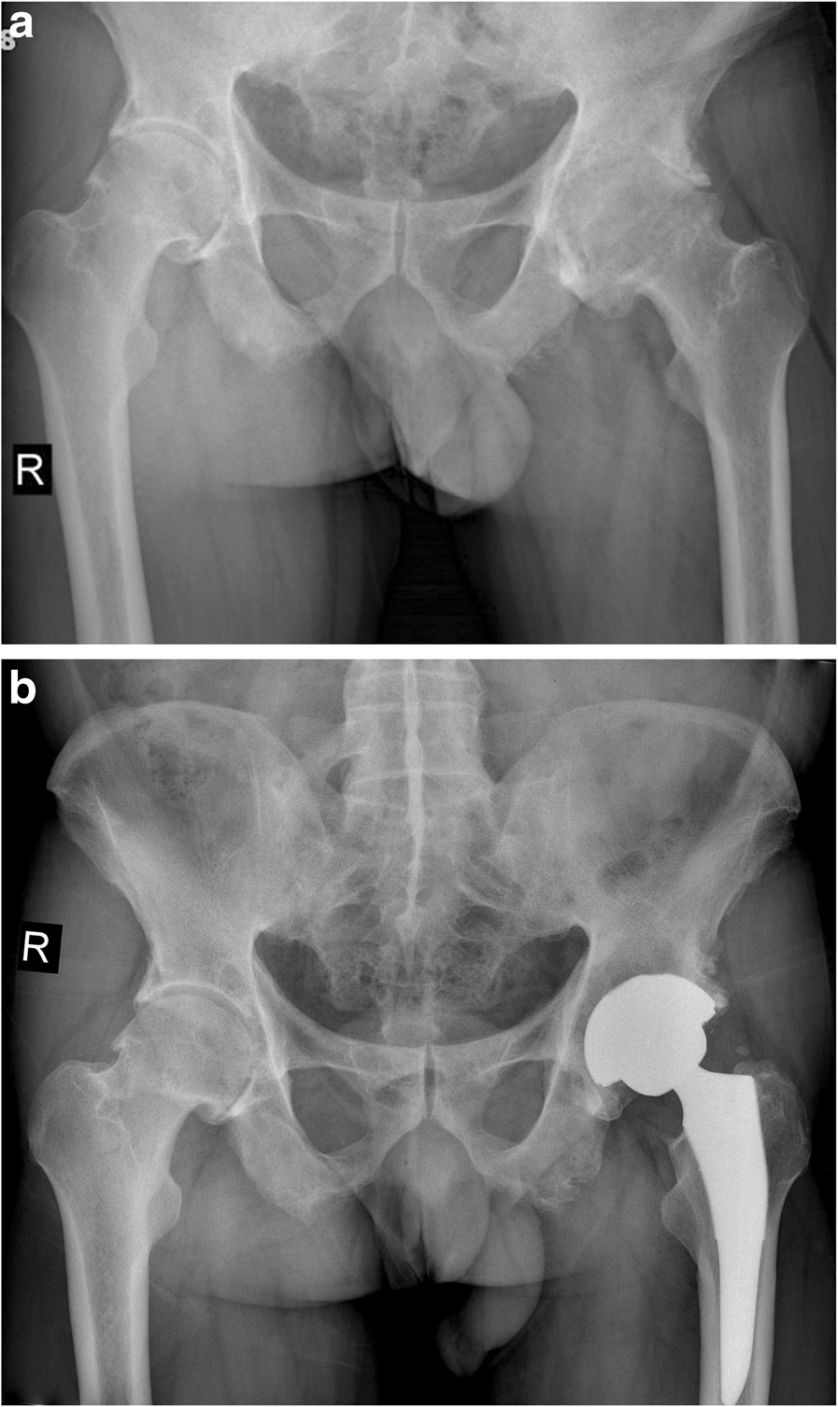
**a** A preoperative radiograph of the hip of a 73 year-old man who had left hip osteoarthritis. **b** A radiograph at 36 month after implantation of short cementless femoral stem showed solid fixation in a satisfactory position

**Fig. 7:**
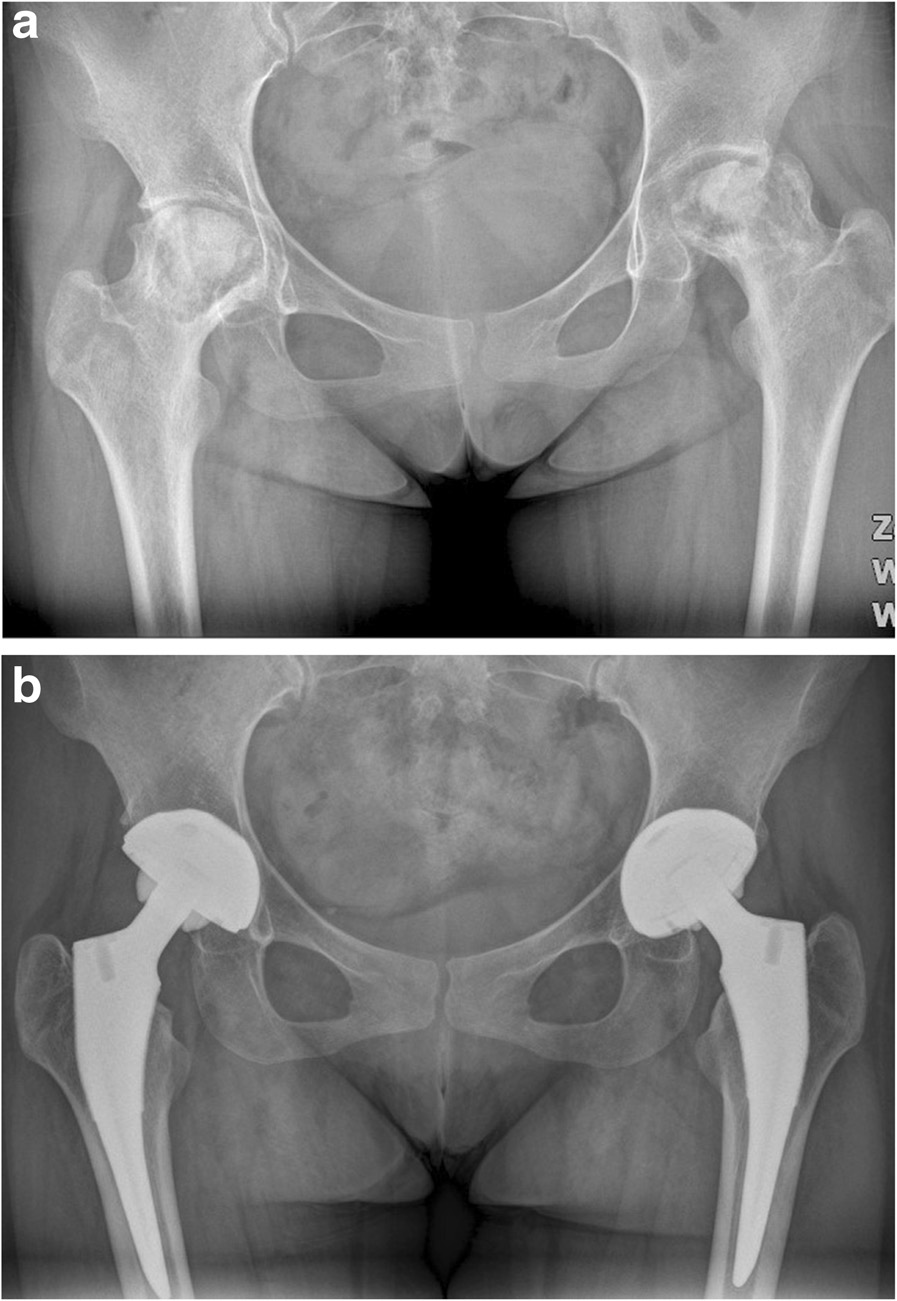
**a** A preoperative radiograph of the hip of a 73-year-old woman who had bilateral femoral head osteonecrosis. **b** A radiograph at 40 months in the right hip and 43 months in the left hip after implantation of short cementless femoral stems showed solid fixation in a satisfactory position

**Fig. 8:**
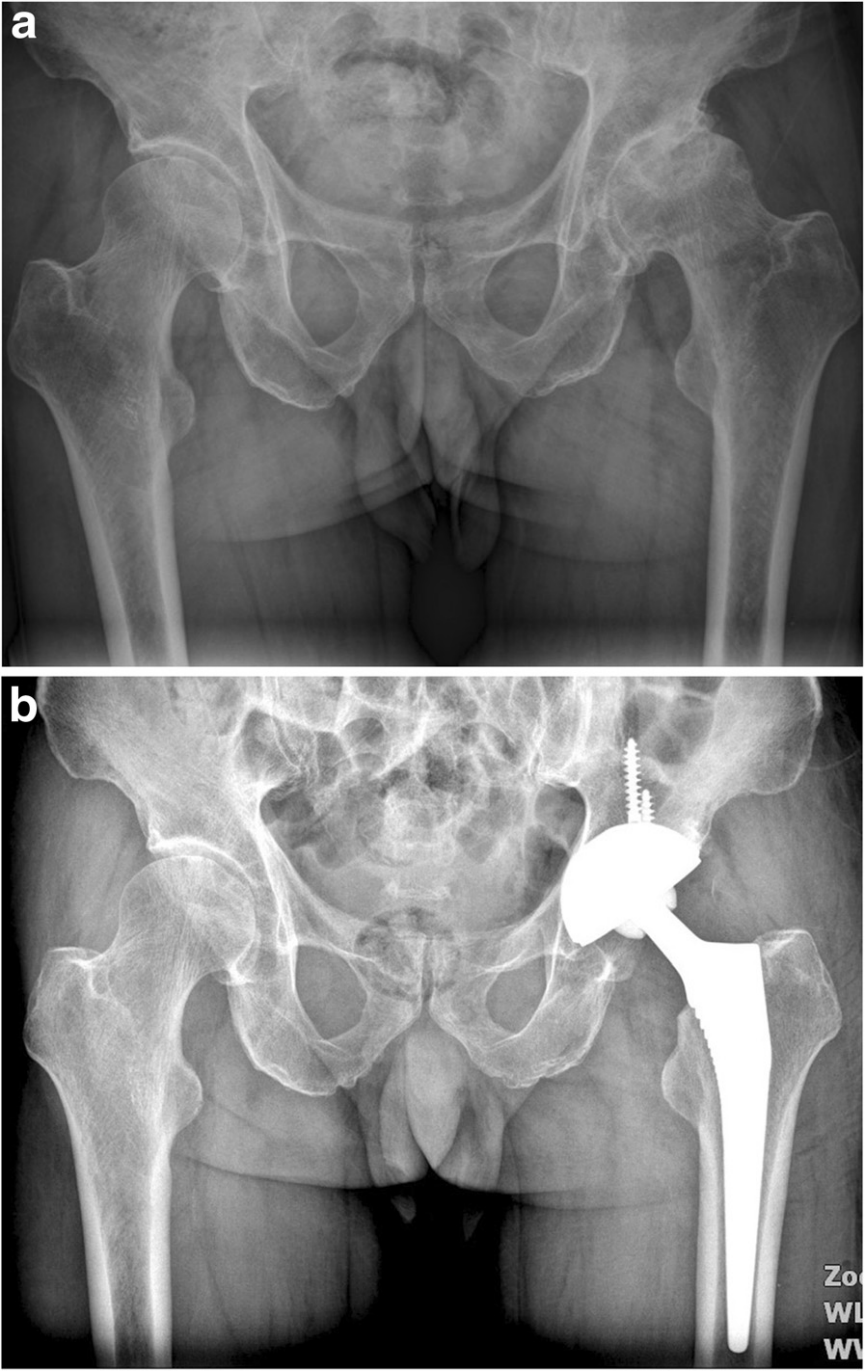
**a** A preoperative radiograph of the hip of a 76-year-old man who had left hip osteoarthritis. **b** A radiograph at 40 months after implantation of conventional cementless femoral stem showed solid fixation in a satisfactory position

**Fig. 9:**
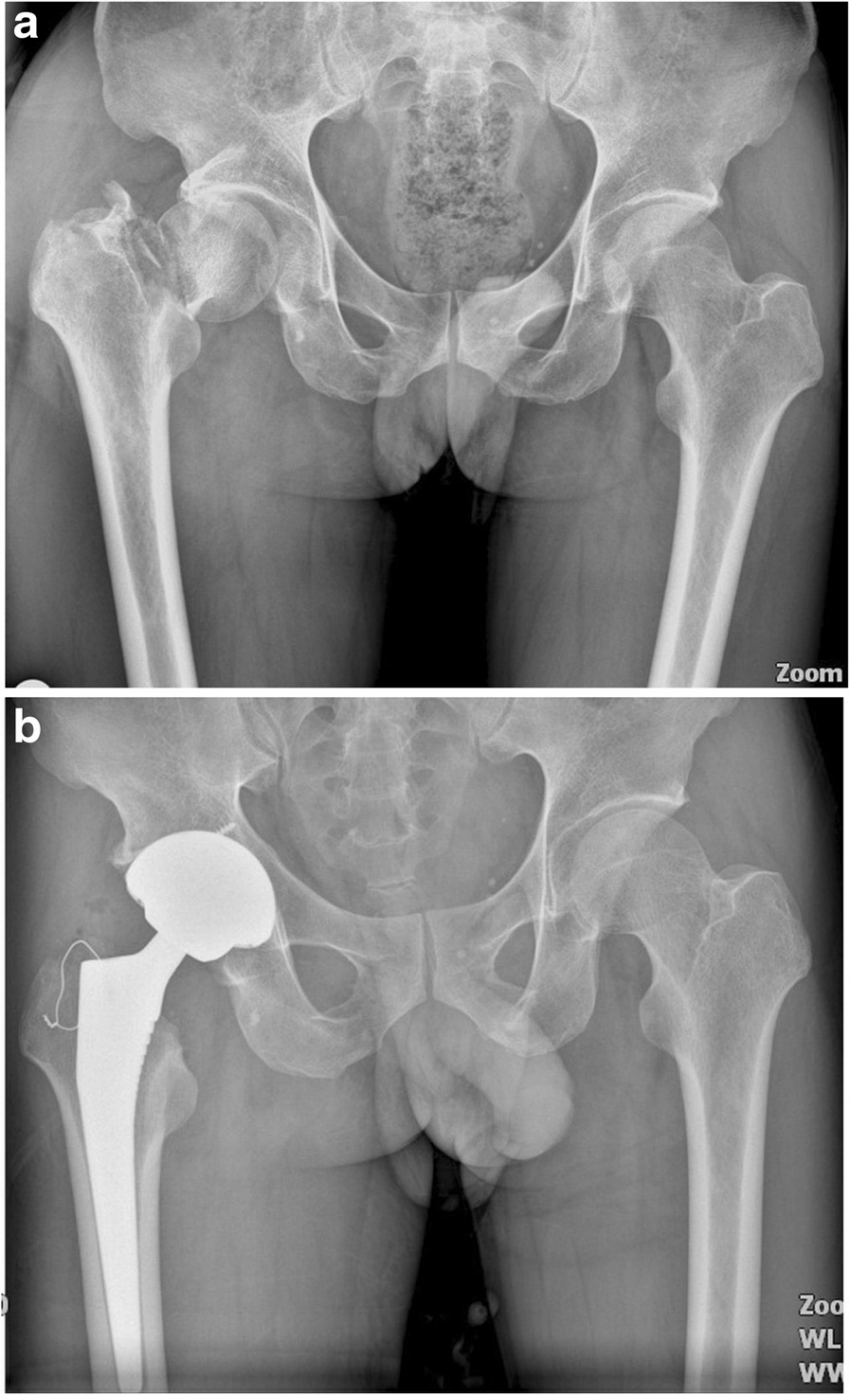
**a** A preoperative radiograph of the right hip of a 75-year-old man who had femoral neck fracture. **b** The patient had an intra-operative fracture and was treated by cerclage wire. A radiography at 42 months showed fracture healed without further complication and conventional cementless femoral stem solid fixation in a satisfactory position

**Fig. 10:**
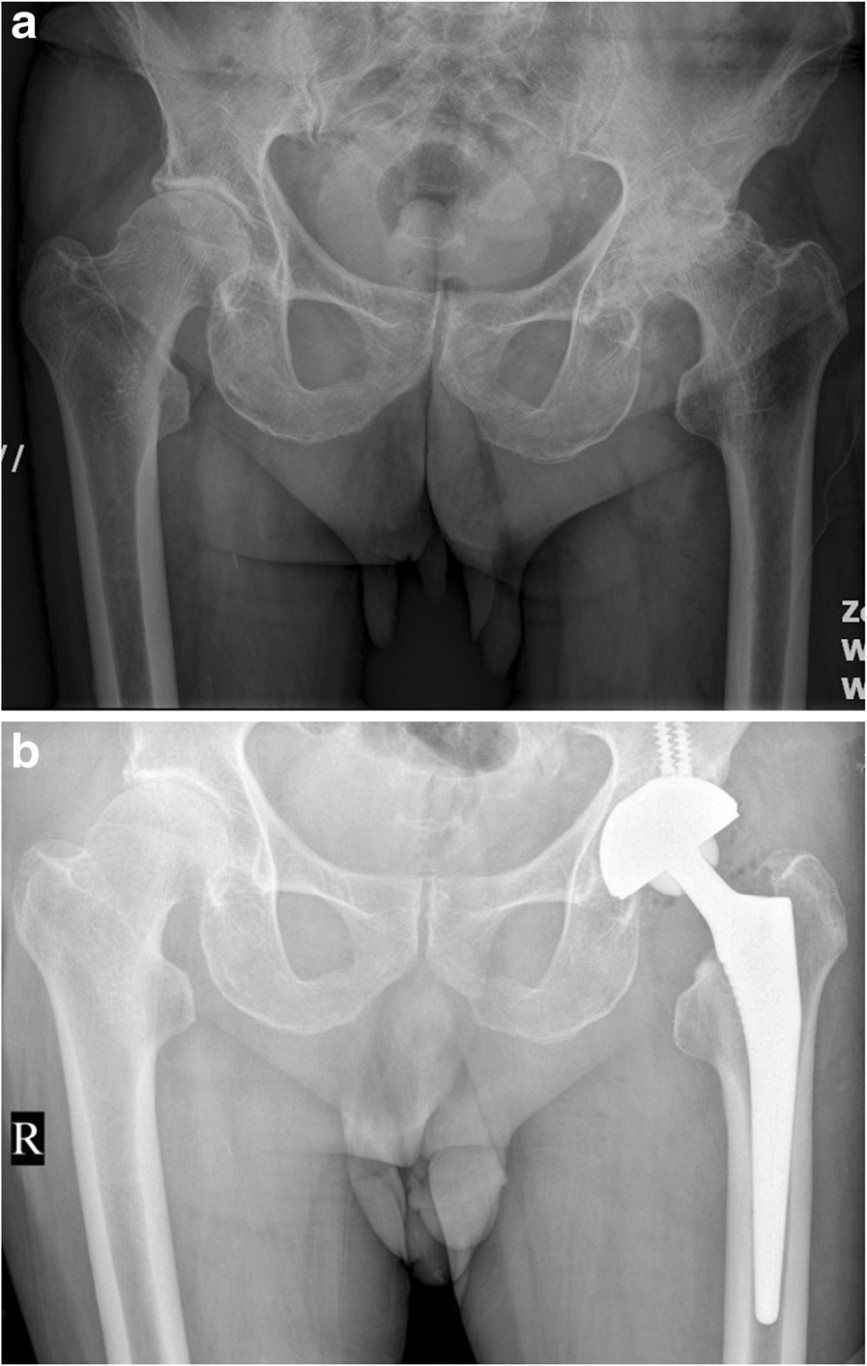
**a** A preoperative radiograph of the hip of a 71-year-old man who had left hip osteoarthritis. **b** A radiograph at 44 months after implantation of conventional cementless femoral stem showed solid fixation in a satisfactory position

**Fig. 11:**
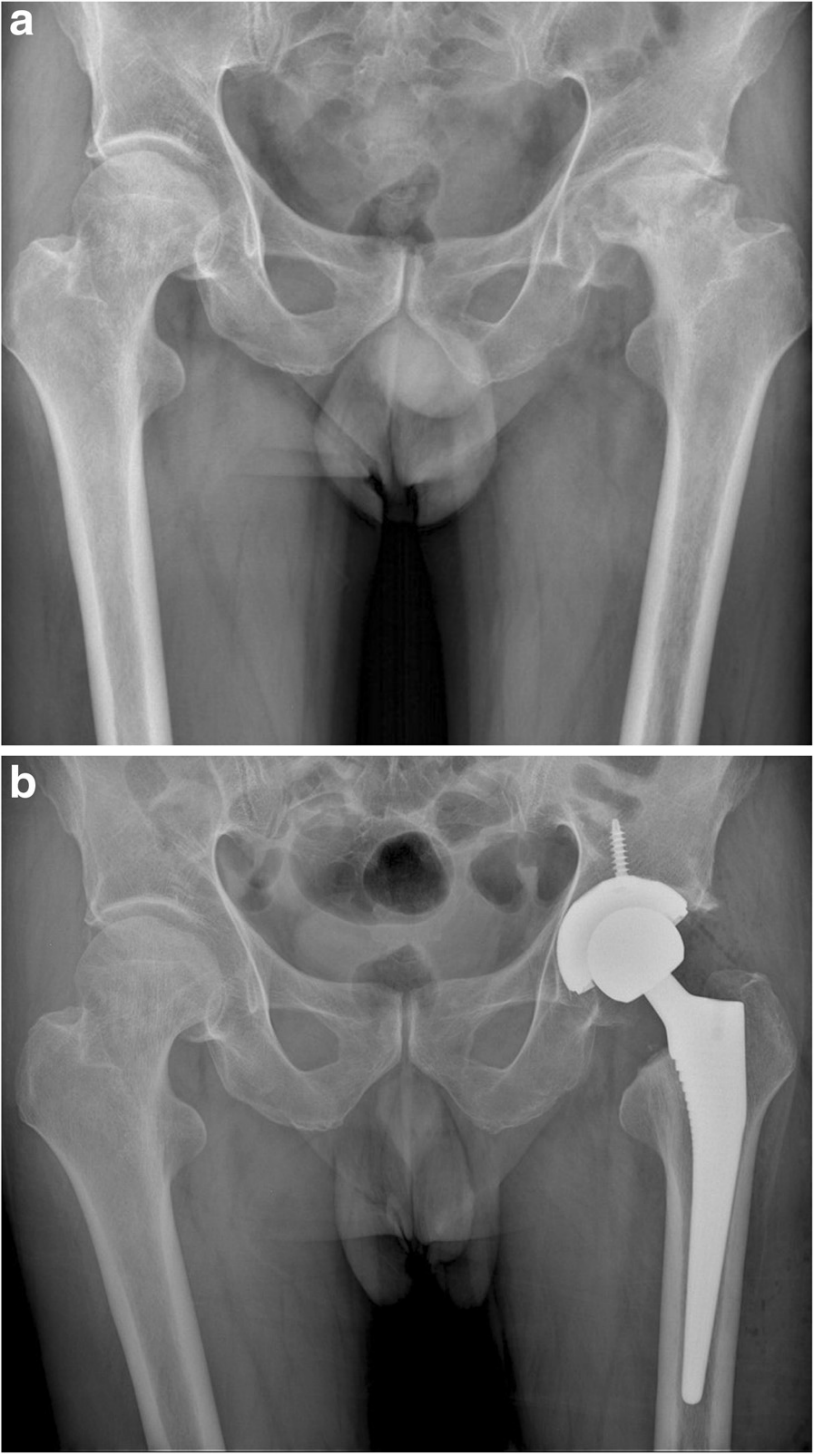
**a** A preoperative radiograph of the hip of a 72-year-old man who had left femoral head osteonecrosis. **b** A radiograph at 42 months after implantation of conventional cementless femoral stem showed solid fixation in a satisfactory position

**Fig. 12:**
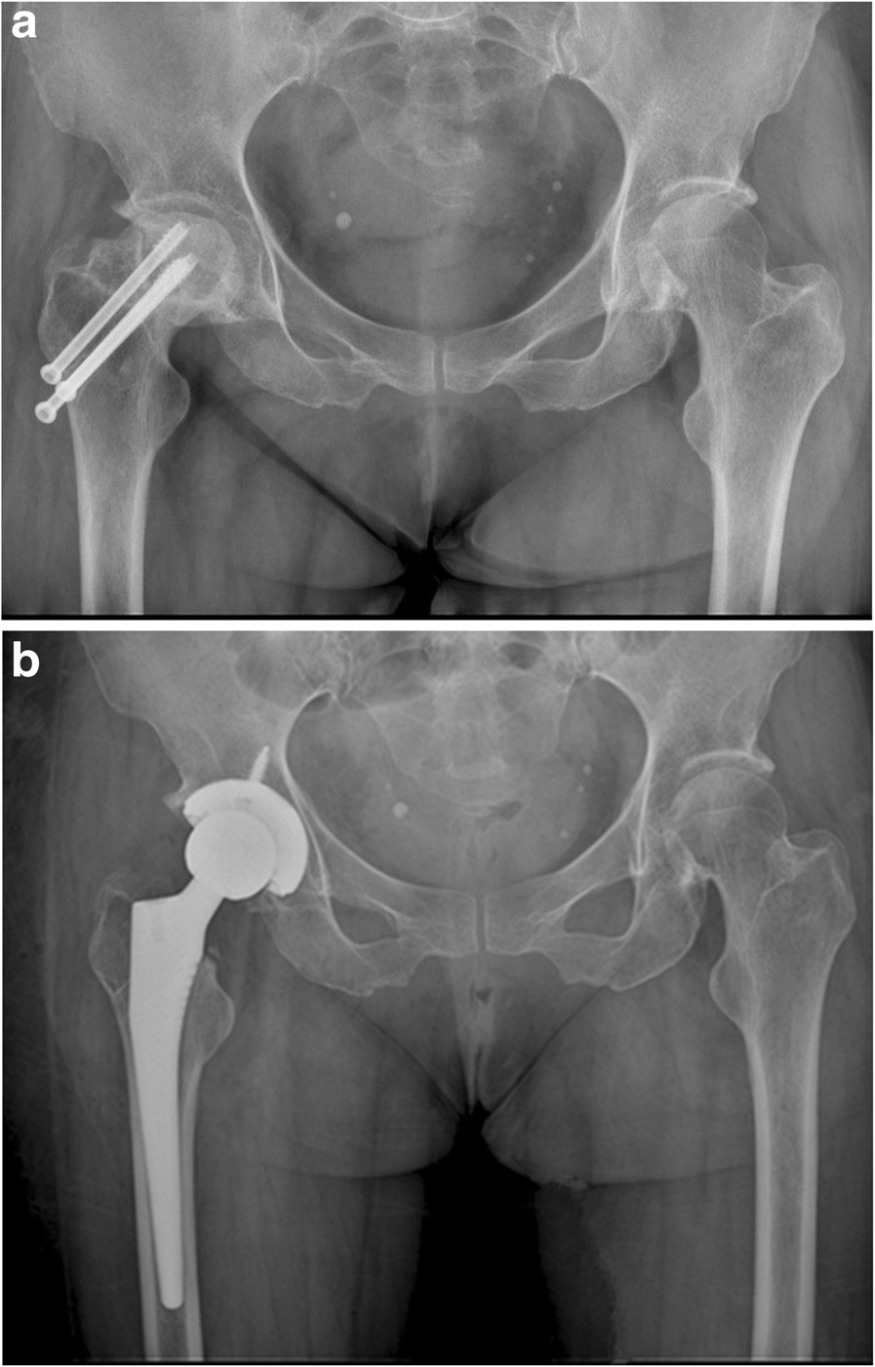
**a** A preoperative radiograph of the hip of a 72-year-old woman who had femoral head osteonecrosis due to femoral neck fracture. **b** A radiograph at 42 months after implantation of conventional cementless femoral stem showed solid fixation in a satisfactory position

There was no statistically significant difference between the two groups in terms of the morphology of the proximal femur, the position of the femoral component, limb-length discrepancy, incidence of the radiolucent line, and prevalence of the migration of the femoral component (Table 3, Figs. 1, 2, 3, 4, 5, 6, 7, 8, 9,10, 11, and 12). Osseo-integration was seen in all the femoral components in both groups. All hips had grade 1 stress shielding in the short-stem group and grade 3 or 4 stress shielding in the conventional stem group.

**Table 3:** Radiogical results of the two stem groups at the final follow-up

Parameters	Short stem	Conventional stem	*P* value
Femoral component position coronal plane			0.921
Neutral	47	48	
Varus	5	6	
Valgus	3	4	
Mean limb-length discrepancy (mm)	46 ± 5.6	50 ± 6.0	0.628
Radiolucent line <1 mm	0	0	
Migration of femoral component <1 mm	0	0

## Discussion

This is the first study to our knowledge comparing the clinical and radiological outcomes between the short femoral cementless stem THA and the conventional femoral cementless stem THA in patients 70 years and older. This study demonstrated that there was no observable difference in HHS and radiographic review level between the two stems. The incidence of thigh pain and intra-operative fracture in the conventional stem group was much higher than in the short stem.

Elderly patients usually have symptoms of osteoporosis, which potentially compromise ingrowth/outgrowth of the implant. Conventional cementless stem has been proved to a good fixation in elderly patients over more than 10-year follow-up. Thus, most orthopedic surgeons may select conventional cementless stem for elderly patients, and they are not sure whether stable fixation can be obtained with the use of the short cementless stem in elderly patients. Stulberg et al. [14] reported no cases of femoral instability in 65 short-stem femoral implants in 60 patients younger than 70 years. Ronak M et al. [15] reported that short stem provide solid, dependable fixation in patients 70 years and older. Y-H Kim et al. [16] also reported that there was no cases of femoral aseptic loosening in short stem in elderly patient with femoral neck fracture. Our study showed there was no short cementless stem that underwent revision for aseptic loosening, migration, subsidence, or osteolysis, and both stems had a good result in elderly patients. Therefore, poor bone quality was not a contraindication, and stable fixation of the short cementless stem was achievable in osteoporotic bones.

The incidence of postoperative thigh pain generally has been higher since the widespread use of cementless femoral stems; however, many authors have reported that short cementless stem has a low incidence rate of thigh pain after THA [7, 17]. Our study showed that the incidence of thigh pain in the conventional stem was significantly higher (10.3 %) than in the short stem. McAuley et al. [18] reported that 8 % of patients have thigh pain postoperatively using conventional stem, which is a similar incidence to our study. Postoperative thigh pain is an interesting phenomenon in THA. It is more commonly associated with well-fixed porous ingrowth or press-fit femoral components. Brook et al. [19] pointed out that patients who have had their stem revised due to persisting thigh pain have exhibited stable in-grown stems at the time of re-operation. Therefore, the high incidence of thigh pain in the conventional stem may be attributed to a tight distal fit of a rigid stem. And the relatively low prevalence of thigh pain in the short stem, we believe, may be result from the axial and torsional stability of the implant and an absence of contact between the distal stem and the femoral cortex.

Intra-operative fracture is another concerned complication in THA, especially elderly patients with osteoporosis. Literatures reported that intra-operative fracture incidence rate usually range from 1 to 6 % [20, 21]. In our study, there was no fracture case in the short stem, however, five fracture cases (8.6 %) in the conventional stem. Though there was no significant difference in intra-operative fracture between two groups, incidence of intra-operative fracture in the conventional stem was higher than the normal range. It demonstrates a decreased risk of intra-operative fracture when short stems are used in the elderly patients. It is likely that short stem is a broach-only system while conventional stem requires both reaming and broaching which leaves fewer bone stock and easily results in high risk of intra-operative fracture in elderly patients.

We acknowledge limitations to our study. First, patients were not randomized because it was the retrospective study design. Second, surgeries were performed by one surgeon in my hospital, all with possible bias in surgical procedure, decision-making, and approaches. Third, conventional cementless THA has greater than 10-year follow-up in the literatures. However, our study is limited by the short-term follow-up in terms of comparing two different stems in patients 70 years and older. It prevents us to draw conclusion about the long-term performance of two stems in the elderly patients. Longer follow-up is under way.

## Conclusions

In conclusion, both short cementless stem and conventional cementless stem provided stable fixation and achieved a satisfactory result in patients 70 years and older and the short cementless stem had a low incidence of thigh pain and intra-operative fracture.

## References

[1] Kim YH (2005). Long-term results of the cementless porous-coated anatomic total hip prosthesis. J Bone Joint Surg (Br).

[2] Ellison B, Berend KR, Lombardi AV, Mallory TH (2006). Tapered titanium porous plasma-sprayed femoral component in patients aged 40 years and younger. J Arthroplasty.

[3] McLaughlin JR, Lee KR (2008). Total hip arthroplasty with an uncemented tapered femoral component. J Bone Joint Surg Am.

[4] Lombardi AV, Berend KR, Mallory TH, Skeels MD, Adams JB (2009). Survivorship of 2000 tapered titanium porous plasma-sprayed femoral components. Clin Orthop Relat Res.

[5] Otani T, Whiteside LA (1992). Failure of cementless fixation of the femoral component in total hip arthroplasty. Orthop Clin North Am.

[6] Molli RG, Lombardi AV, Berend KR, Adams JB, Sneller MA (2012). A short tapered stem reduces intraoperative complications in primary total hip arthroplasty. Clin Orthop Relat Res.

[7] Santori FS, Santori N (2010). Mid-term results of a custom-made short proximal loading femoral component. J Bone Joint Surg (Br).

[8] Dorr LD, Faugere MC, Mackel AM, Gruen TA, Bognar B, Malluche HH (1993). Structural and cellular assessment of bone quality of proximal femur. Bone.

[9] Harris WH (1969). Traumatic arthritis of the hip after dislocation and acetabular fractures: treatment by mold arthroplasty. An end-result study using a new method of result evaluation. J Bone Joint Surg Am.

[10] Barrack RL, Paprosky W, Butler RA, Palafox A, Szuszczewicz E, Myers L (2000). Patients’ perception of pain after total hip arthroplasty. J Arthroplasty.

[11] Kim YH, Kim VE (1993). Early migration of uncemented porous coated anatomic femoral component related to aseptic loosening. Clin Orthop Relat Res.

[12] Engh CA, Massin P, Suthers KE (1990). Roentgenographic assessment of the biologic fixation of porous-surfaced femoral components. Clin Orthop Relat Res.

[13] Engh CA, Bobyn JD (1988). The influence of stem size and extent of porous coating on femoral bone resorption after primary cementless hip arthroplasty. Clin Orthop Relat Res.

[14] Stulberg SD, Dolan M (2008). The short stem: a thinking man’s alternative to surface replacement. Orthopedics.

[15] Patel RM, Smith MC, Woodward CC, Stulberg SD (2012). Stable fixation of short-stem femoral implants in patients 70 years and older. Clin Orthop Relat Res.

[16] Kim YH, Oh JH (2012). A comparison of a conventional versus a short, anatomical metaphyseal-fitting cementless femoral stem in the treatment of patients with a fracture of the femoral neck. J Bone Joint Surg (Br).

[17] Kim YH, Park JW, Kim JS (2013). Behaviour of the ultra-short anatomic cementless femoral stem in young and elderly patients. Int Orthop.

[18] McAuley JP, Moore KD, Culpepper WJ, Engh CA (1998). Total hip arthroplasty with porous-coated prostheses fixed without cement in patients who are sixty-five years of age or older. J Bone Joint Surg Am.

[19] Brown TE, Larson B, Shen F, Moskal JT (2002). Thigh pain after cementless total hip arthroplasty: evaluation and management. J Am Acad Orthop Surg.

[20] Kavanagh BF (1992). Femoral fractures associated with total hip arthroplasty. Orthop Clin North Am.

[21] Lindahl H (2007). Epidemiology of periprosthetic femur fracture around a total hip arthroplasty. Injury.

